# Hospital Meetings

**Published:** 1902-12-13

**Authors:** 


### HOSPITAL MEETINGS.

### ALTERATIONS AT THE LONDON HOSPITAL.

This quarter (Wednesday, 3rd inst.), for the first time, the governors of the London Hospital were able to meet in the new Board Room, a demonstration, as the Hon. Sydney Holland (chairman) remarked, of the improvements being effected. The old Board Room, he explained, had become inadequate to the present needs of the hospital, and every meeting in it dislocated the work of the secretary's office to a considerable extent, since the accommodation was so cramped that the committee-room had to be used as an office. The old room will now be devoted entirely to office purposes, and the work will not be subject to interruptions.

### The New Out-Patients' Department.

The new out-patients' department is practically finished, and Mr. Holland said the governors might congratulate themselves on the fact that the building would not only serve to satisfy the general needs of the East End of London, but would also be a type or model on which other out-patient departments would probably be constructed. It is so arranged that two complete sets of rooms at one end are devoted entirely to surgical cases, while at the other end is a large hall with seating accommodation for 900 people. Full provision has been made for the registration office, and for the dispensary department. The latter will afford ample provision for installing a complete manufacturing plant in connection with the hospital work, and thereby considerable economy will be secured, for all ointments, pills, decoctions, etc., needed in the hospital will be manufactured there. On the upper floor there is special provision for the throat, nose, and ear department, for massage cases, dental cases, and the diseases of women, orthopædic cases, and cases of skin disease. On the top floor, new accommodation has been found for Queen Alexandra's Finsen Light Treatment of lupus, now carried out at the end of the temporary buildings in the hospital garden. On this floor will also be accommodation for the medico-electro-therapeutic treatment, including all the work done by means of the Rœntgen rays, and for a large and ample ophthalmic department.

It has been found necessary to increase the number of out-patient officers. Two have therefore been added to the staff of four, and they will in future have additional work as senior clinical assistants in this department, so that they will not only see cases in the receiving-room, but will also act as assistants to the consulting physicians and surgeons when seeing those cases referred from the receiving-room to the out-patients' department. This arrangement, said Mr. Holland, will add considerably to the efficiency of the work, and will also make the appointments more valuable to young medical men. It adds, however, to the increasing cost of upkeep.

Arrangements have been made for using this building on December 11th, and next year their Majesties the King and Queen have most graciously consented to visit the hospital and formally open this wing.

### Other Departments.

In the main corridor great progress has been made, and excellent accommodation has been provided in the new receiving-room. The remodelling of the old wing is in full progress. The old lobbies leading to Gloucester, Sophia and Talbot wards have been gutted, and the foundations have been put in for a new staircase, corresponding with that already in use in the West wing. The rooms in the old part of the centre block are almost completed, and Sophia ward on the first floor of the old wing is in occupation.

Good progress has been made with the new laundry, on the site of the old John Baker's Almshouses, and as soon as this is completed the laundry will be moved from its present position in the basement of the old east wing, and this will afford improved accommodation for the porters, and will prove an inestimable boon to patients in the accident and surgical wards above. The new theatres are giving great satisfaction.

### The Hospital and Public Bodies.

Negotiations are going on between the London County Council and the hospital with respect to the building of a Coroner's Court on the premises. The committee have long recognised the necessity of providing better accommodation for the coroner; that at present in use was never intended to be permanent. The difficulty of finding a position has been great. Now, however, the old surgical out-patients' department offers a suitable one, and it is hoped that the negotiations may be so far satisfactory as to enable the committee to carry out this improvement.

The site which the School Board for London purchased from the governors in Myrdle Street has now been transferred to them, and the committee view with some alarm the statement in a recent issue of the *Times* that three more sites on the hospital estate have been scheduled by the School Board. They hope, however, that such pressure may be brought to bear that the Board may be prevented from going further in the matter, especially as they already occupy three large sites on the hospital estate.

### New Hebrew Wards.

As soon as the new out-patients' department is open, opportunity offers for the west wing to be dealt with, and after careful consideration it has been determined that new Hebrew wards shall be built, in which medical and surgical cases, and male and female, may be separated. The total cost is estimated at £32,000.

### The Nurses.

"Good progress," said Mr. Holland, "has been made in providing a site for the new nurses' home, which is to be built on the land adjoining the site of the new laundry. It is expected that before the next meeting of the governors we shall have been able to put this in hand and to have begun the work. It is at present difficult to find room for the nurses, many of whom are most inconveniently lodged in small houses in the neighbourhood of the hospital."

### The Hospital and King Edward's Fund.

A full statement of the financial position of the hospital, the speaker went on, was submitted to the council of King Edward's Hospital Fund, and in addition to the £5,000 a year already granted, they had given a further sum of £3,500 for this year. But the whole of this £8,500 is but a small portion of the £120,000, including £87,000 for upkeep, that is necessary each year to maintain the efficiency of the hospital, and he was disappointed that no more had been given.* It was not well to complain when a large sum was received, but he could not help comparing it with what other hospitals got. Twice the work of any other London hospital (with one exception) was being done by the London, and he felt that the special circumstances had not been considered. The hospital was not, as many people seemed to imagine, in a strong financial position; in fact, he did not think there was a hospital in London worse off. Only £20,000 came from investments; the cost of upkeep would soon be £100,000 a year, and £80,000 had to be obtained from the public. The hospital was committed to an expenditure of £100,000 on improvements before the necessary conditions of the yearly grant of £5,000 could be kept; improvements had been wanted for years, but because it fell to this generation to carry them out the burden was great. Next year would be the Quinquennial Appeal year, and the committee most earnestly asked all the Governors to do their very utmost to further the success of the appeal. Personal application was the only way, since there were so many appeals before the public. Undoubtedly the visit of the King and Queen would help to bring the hospital before the public to some extent, and the Lord Mayor had also promised to give a Mansion House dinner; but however important these two events would be, they would only supplement the individual efforts on which the committee mainly depended.

### The Convalescent Home.

A full year's work was completed at the Hermann de Stern Convalescent Home, for which the magnificent sum of £50,000 to endow and £10,000 to build had been given by the executors of the late Baron de Stern. What he (the Chairman) thought was a perfect convalescent home had been opened at Felixstowe. At the end of the year there was a debt of £600; this, however, had been paid off by the generosity of Baron de Stern and Mr. R. L. E. Raphael.

He thought Mr. Rowland Plumbe, the architect, should be congratulated on having enabled the committee to carry out all the improvements without closing any beds. Efforts were being made to obtain an endowment for the medical college. Legacies had been received for the hospital to the amount of only £3,869, and donations £324. It had been a bad year.

### Working at High Pressure.

Sir Edmund Hay Currie seconded the adoption of the report. He congratulated the hospital on the new quarters. There was a ring of feeling in the Chairman's speech which seemed to denote that he thought the hospital was not sufficiently cared for by the outside public, and it was evident that he thought King Edward's Fund might have given a far larger amount. As secretary of the Hospital Sunday Fund, it was his (the speaker's) business to know the financial state of all the hospitals in London, and he was able to say that with one exception the London Hospital was doing what looked on paper equal to the work of any two hospitals in London. The difference between the London Hospital and others was that the former was always at high pressure; anyone might see this on entering; there was a life and "go" which was not necessary elsewhere. A small hospital could not possibly do the work accomplished by a large one, and he wondered how this hospital had managed to go on for so long without the improvements now being carried out. Not only was there twice as much work as in any other (with one exception), but the London was also the largest children's hospital in London. All the special departments were represented, with the result that the staff was unparalleled. Alluding to a map recently published in the daily press showing how the London hospitals tended to be congested in the Strand neighbourhood, Sir Edmund Currie said the London Hospital was just where it was most wanted—in the heart of a gigantic population depending upon it for help in sickness and accident—and it would be impossible ever to close its doors.

In reply to a Governor, who regretted the condition attached to the yearly grant of £5,000 from King Edward's Fund, Mr. Holland said he could not express his gratitude that that condition had been made, otherwise the committee would never have had the pluck to start on the improvements. The same gentleman having inquired what facilities there would be for the Governors to view the ceremony of the opening, the Chairman said his own inclination was to try and turn the occasion to profit by letting seats.

The report was adopted, and the perennial joke of the Chairman, who did not fail to propose his own vote of thanks, received its customary meed of mirth.

* This statement, and the figures which follow, do not tally with any printed account or official documents yet issued by the London Hospital. It would appear that either our reporter or Mr. Sydney Holland has fallen into error. The task before the London Hospital is so difficult that wrong figures must make it impossible of accomplishment.—Ed. The Hospital.

